# Beyond Blood Pressure: Arterial Stiffness as a Hemodynamic and Neuroadrenergic Axis Linking Hypertension, Cardiac Remodeling, and Heart Failure

**DOI:** 10.3390/life16040682

**Published:** 2026-04-16

**Authors:** Pasquale Ambrosino, Cesare Cuspidi, Claudio Candia, Christian Basile, Mauro Maniscalco, Guido Grassi

**Affiliations:** 1 Istituti Clinici Scientifici Maugeri IRCCS, Scientific Directorate of Telese Terme Institute, 82037 Telese Terme, Italy; 2 Department of Medicine and Surgery, Milano-Bicocca University, 20126 Milan, Italy; cesare.cuspidi@unimib.it; 3 Istituti Clinici Scientifici Maugeri IRCCS, Pulmonary Rehabilitation Unit of Telese Terme Institute, 82037 Telese Terme, Italy; claudio.candia@icsmaugeri.it (C.C.); mauro.maniscalco@icsmaugeri.it (M.M.); 4 Department of Clinical Science and Education, Karolinska Institutet, 17177 Stockholm, Sweden; christian.basile@ki.se; 5 ANMCO Research Center, Heart Care Foundation, 50121 Florence, Italy; 6 Department of Advanced Biomedical Sciences, Federico II University, 80131 Naples, Italy

**Keywords:** arterial stiffness, hypertension, heart failure, ventricular–vascular coupling, sympathetic activation, cardiovascular phenotyping, disability, exercise, rehabilitation

## Abstract

Arterial stiffness has traditionally been interpreted as a marker of vascular ageing and cumulative blood pressure exposure. Increasing evidence, however, indicates that it should be viewed as an active determinant of cardiovascular loading conditions rather than a passive epiphenomenon. By accelerating pulse wave velocity and altering the timing of wave reflection, large artery stiffening increases central systolic pressure, augments late systolic load, and facilitates the transmission of pulsatile energy to the microcirculation. These hemodynamic alterations shape ventricular remodeling, influence ventricular–vascular coupling, and contribute to organ vulnerability even when brachial blood pressure appears adequately controlled. In this review, population-based observations and mechanistic human studies are integrated to position arterial stiffness as a stage-dependent dimension of cardiovascular disease. Community data illustrate its association with different blood pressure phenotypes and early cardiac structural changes, whereas evidence from advanced heart failure settings helps contextualize arterial stiffness within states of marked autonomic activation. Taken together, this perspective suggests that arterial stiffness is not merely a marker of cumulative damage, but a mediator that contributes to disease progression across clinical stages and, in practical terms, a phenotyping dimension along the trajectory from hypertension to heart failure.

## 1. Introduction

Over the last two decades, arterial stiffness has progressively moved from being regarded as a descriptive feature of vascular ageing to a clinically meaningful dimension of cardiovascular disease [1,2]. This shift reflects a broader change in how cardiovascular risk is conceptualized, with growing attention paid to arterial properties and pulsatile hemodynamics rather than to blood pressure values alone [1,3]. Indeed, patients with the same blood pressure readings measured in the brachial artery may show significant differences in cardiac structure, vascular damage, and outcomes [4]. These discrepancies are now considered clinically relevant rather than anecdotal, since they challenge the common perception that blood pressure values are the only determinants of the cardiovascular stress burden.

Arterial stiffness represents, within a measurable property of the arterial wall, the cumulative effects of ageing, cardiovascular risk factors, and chronic hemodynamic stress [1,3]. Its clinical appeal has expanded alongside the development of reliable noninvasive techniques [2], while its relevance extends beyond risk stratification. Arterial stiffness does not simply reflect blood pressure values but modifies how pressure is transmitted and perceived by the heart and the microcirculation over time [5]. As a result, arterial stiffening may already be present in patients who, based on conventional cuff measurements, appear well controlled [6].

The present review follows a continuous pathophysiological trajectory from uncomplicated hypertension to overt heart failure, placing arterial stiffness at the center of this sequence. Rather than presenting new analyses, it integrates evidence from population-based cohorts with mechanistic human studies to construct a coherent interpretative framework. The underlying premise is that arterial stiffness represents a cross-sectional vascular property that also carries longitudinal information, reflecting where an individual lies along the disease trajectory from early hypertension to advanced heart failure. Within this context, observations from the Pressioni Arteriose Monitorate e Loro Associazioni (PAMELA) study are used to illustrate how arterial stiffness relates to blood pressure phenotypes and early cardiac remodeling in the community [4]. Data derived from the Baroreflex Activation Therapy for Heart Failure (BATHF) program are then considered to explore the interaction between arterial stiffness and sympathetic neural activation in advanced stages of disease [7,8]. By integrating these apparently distant clinical settings through original conceptual schematics specifically developed for this manuscript, the review aims to frame arterial stiffness as a phenotyping dimension, namely a continuous, measurable vascular property that reflects structural, hemodynamic, and autonomic information from uncomplicated hypertension to advanced heart failure. Unlike conventional phenotyping frameworks that classify patients by ejection fraction, blood pressure pattern, or comorbidity profile, arterial stiffness holds the promise of capturing the cumulative vascular exposure and the current functional state of the arterial wall within a single parameter.

## 2. Scope and Search Strategy

This is a narrative review. The literature was identified through targeted searches of PubMed/MEDLINE, Scopus, and Web of Science, combining terms related to arterial stiffness, pulse wave velocity (PWV), cardio-ankle vascular index (CAVI), ventricular–vascular coupling, hypertension, heart failure, and sympathetic activation. Reference lists of the selected articles were also examined to identify additional relevant studies. Eligible publications were required to involve adult human populations and to provide original data, quantitative synthesis, or authoritative consensus on arterial stiffness and its relationships with blood pressure phenotypes, cardiac structure, ventricular–vascular coupling, or autonomic activation. Only articles published in English and indexed in the consulted databases were considered. Within this framework, priority was assigned, in descending order, to international consensus documents and guidelines, systematic reviews and meta-analyses, large prospective cohort studies, and mechanistic investigations in humans using direct measures of sympathetic neural activity or validated vascular indices. Particular attention was given to publications from the past five years, although earlier landmark studies were retained whenever necessary to preserve historical context or mechanistic continuity. Conference abstracts, editorials without original content, case reports, and purely preclinical studies without clear human relevance were excluded. When more than one publication addressed the same research question using overlapping datasets, the most recent and methodologically robust report was selected. In addition, the reference lists of all retrieved articles were screened to capture relevant studies that had not emerged from the database search.

The review is structured around two complementary datasets that anchor the pathophysiological narrative at opposite ends of the cardiovascular disease spectrum. In particular, the PAMELA cohort provides population-level evidence on arterial stiffness across blood pressure phenotypes and its association with early cardiac structural changes [4]. The BATHF program offers a clinical model in which arterial properties, left ventricular systolic function, and muscle sympathetic nerve activity were simultaneously assessed in patients with advanced heart failure and reduced ejection fraction [7,8]. To contextualize these findings within the broader literature, data from independent cohorts and recent meta-analyses have been integrated throughout the manuscript, and methodological differences across arterial stiffness indices are addressed where relevant. Supplementary Table S1 maps each figure to its primary data source and specifies the element of original visual synthesis contributed by this manuscript.

## 3. Arterial Stiffness in Hypertension: Measurement and Pathophysiological Framing

Arterial stiffness arises from the interaction between structural and functional components of the arterial wall [9]. Structural alterations, including elastin fragmentation, collagen accumulation, calcification, and extracellular matrix remodeling, are driven by aging, cardiovascular risk factors, inflammation, and metabolic disorders [3]. Functional factors, including vascular smooth muscle tone and endothelial function, modulate stiffness over shorter time scales, contributing to its dynamic and variable nature [10]. As a result, arterial stiffness cannot be fully inferred from a single resting blood pressure measurement. Indices that are highly pressure-dependent tend to reflect reversible functional components, whereas pressure-independent measures better capture cumulative structural damage [5]. This distinction is particularly important across heterogeneous blood pressure phenotypes, where mean pressure may differ, but cumulative pulsatile stress may be similar [1], supporting the view of arterial stiffness as an integrative exposure rather than a static parameter. The clinical relevance of arterial stiffness is closely linked to the distinction between steady and pulsatile arterial load [10]. In brief, steady load refers to the non-pulsatile component of arterial pressure, best represented by mean arterial pressure, which depends primarily on cardiac output and total peripheral resistance [10]. Pulsatile load refers to the oscillatory component, driven by arterial compliance, PWV, and the timing and magnitude of wave reflections. As arteries stiffen, the balance shifts toward a greater pulsatile fraction, increasing the intermittent mechanical stress imposed on the left ventricle and the microcirculation even when mean pressure remains stable [3]. This explains the dissociation between brachial blood pressure and target organ damage often observed in epidemiological studies [11], and underscores the limitations of relying on cuff-based blood pressure alone for cardiovascular risk stratification [12].

Wave reflections are central to these effects [10]. In compliant arteries, reflected waves return during diastole and support coronary perfusion, whereas increased stiffness accelerates wave return into systole, raising systolic pressure and lowering diastolic pressure [13]. This hemodynamic pattern increases myocardial oxygen demand and may impair subendocardial perfusion [9], promoting concentric remodeling and diastolic dysfunction that precede overt systolic impairment in hypertensive heart disease [14].

Excess pulsatility also affects the microcirculation [15], particularly in low-resistance organs such as the kidney and brain. Reduced buffering by stiffened arteries allows greater pressure transmission to the microvasculature, contributing to microvascular damage and indirectly to cardiac dysfunction through cardiorenal and neurovascular pathways [1]. In population studies, these extracardiac effects often coexist with early cardiac structural changes [11].

Arterial stiffness should therefore be considered a dynamic property [16]. Acute changes in sympathetic activity, endothelial function, and vasoactive mediators can transiently alter stiffness, while long-term structural changes define its baseline [9]. This dual nature explains why stiffness may correlate weakly with simple autonomic markers in population studies but strongly with direct measures of sympathetic activity in advanced disease [7], a distinction that becomes increasingly relevant across stages of the cardiovascular continuum.

The growing interest in arterial stiffness has led to diverse measurement techniques [2,16], which differ in their physiological targets and interpretive implications. Recognizing these differences is essential, particularly in population studies where stiffness indices are used to assess cardiovascular risk across varied blood pressure phenotypes [11,16]. Carotid–femoral PWV is the gold standard for assessing aortic stiffness [16], closely reflecting arterial wall structure [2] and recently associated with short- and mid-term mortality in acutely ill medical patients in the emergency setting [17]. Peripheral indices such as CAVI assess longer arterial segments and aim to reduce dependence on blood pressure at the time of measurement [5]. Although not interchangeable, these methods provide complementary information and help distinguish cardiovascular risk among individuals with similar blood pressure levels [12].

It should be noted, however, that these indices are not interchangeable. Carotid–femoral PWV and CAVI differ in the arterial segments assessed, in their dependence on blood pressure at the time of measurement, and in the amount of prognostic data supporting their use. Recent international recommendations have emphasized the need for device-specific validation and have cautioned against treating derived indices as equivalent to carotid-femoral PWV in clinical decision-making [18]. Similarly, an expert consensus on the clinical application of PWV has provided practical guidance for integrating stiffness measurement into cardiovascular risk assessment, while acknowledging that the evidence base remains largely confined to specific populations [19].

Overall, arterial stiffness should be viewed not merely as a marker of vascular aging or cumulative blood pressure exposure [3] but as a phenotyping dimension that reflects arterial structure, vascular tone, pulsatile hemodynamics, and end-organ vulnerability. This framework clarifies how arterial stiffening interacts with blood pressure phenotypes and early cardiac remodeling in the general population.

## 4. Evidence from the PAMELA Cohort: Arterial Stiffness Across Blood Pressure Phenotypes and Early Cardiac Remodeling

Community-based cohorts offer a valuable setting in which arterial stiffness can be interpreted as a modifier of cardiovascular phenotypes rather than as an isolated marker. The PAMELA study exemplifies this approach by combining clinic, home, and 24 h ambulatory blood pressure measurements with echocardiographic assessment in a representative sample of approximately 3200 individuals, followed over several decades [4,20]. This design allows arterial stiffness to be examined in individuals who are frequently encountered in routine clinical practice, often before the development of overt cardiovascular disease [21]. Within this cohort, arterial stiffness was assessed using the cardio-ankle vascular index, a parameter designed to reflect large artery stiffness with limited dependence on concurrent blood pressure [22], and was recently shown to track nocturnal blood pressure patterns and left ventricular mass in the same population [23].

**Figure 1:**
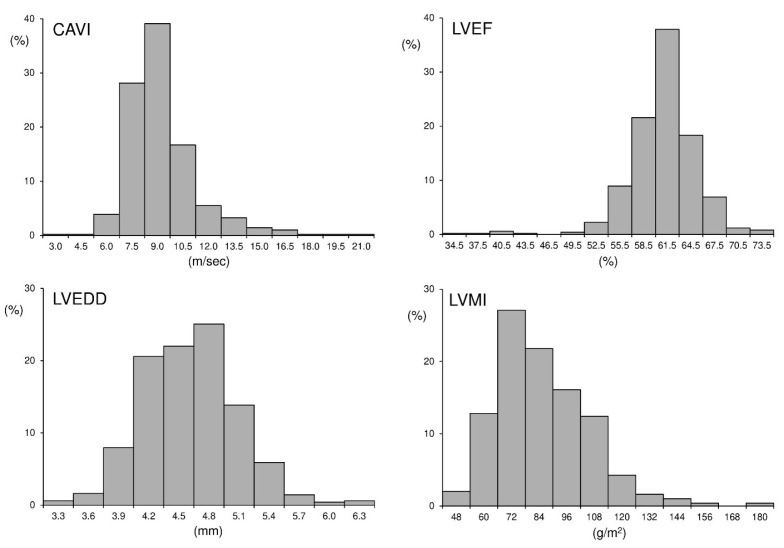
Distribution of arterial stiffness and echocardiographic parameters in the PAMELA cohort. The figure shows the distribution of cardio-ankle vascular index (CAVI), left ventricular ejection fraction (LVEF), left ventricular end-diastolic diameter (LVEDD), and left ventricular mass index (LVMI). Distributions refer to the overall study population, including both normotensive and hypertensive individuals. The panels were originally conceived and designed for this manuscript to provide a joint visualization of parameters that were reported separately in the PAMELA study (Cuspidi et al. *Clinical* *Research in Cardiology*, 2024 [23]). No previously published figure combines these distributions within a single comparative layout.

Figure 1 illustrates the distribution of arterial stiffness alongside key echocardiographic parameters in normotensive and hypertensive individuals, based on data from a recent PAMELA analysis [23]. Increased stiffness is already detectable in hypertensive participants despite largely preserved left ventricular systolic function [24]. Structural indices such as left ventricular mass and chamber dimensions also show early deviations, suggesting that vascular alterations may accompany the early stages of cardiac remodeling before overt systolic dysfunction becomes apparent [25]. This pattern is consistent with the notion that vascular alterations may precede, rather than simply accompany, clinically manifest cardiac impairment [26].

**Figure 2:**
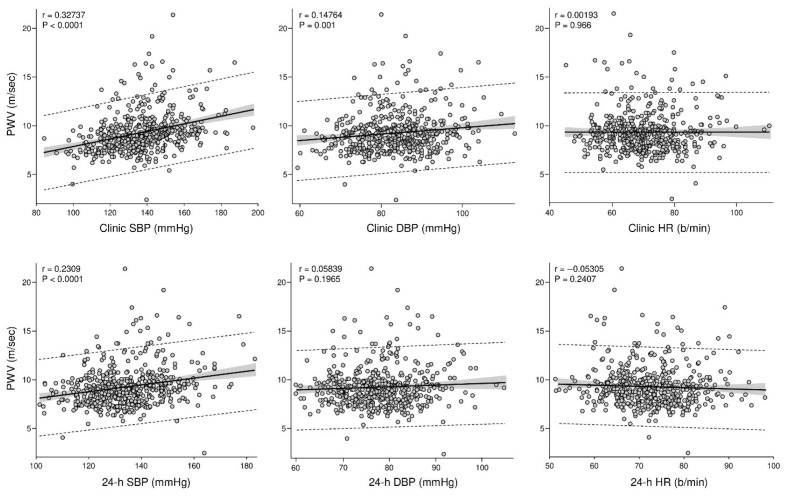
Relationships between arterial stiffness, blood pressure components, and heart rate in the PAMELA cohort. Scatter plots illustrate the associations between cardio-ankle vascular index (CAVI) and clinic systolic blood pressure, clinic diastolic blood pressure, and clinic heart rate (upper panels), as well as 24 h systolic blood pressure, 24 h diastolic blood pressure, and 24 h heart rate measured by ambulatory monitoring (lower panels). Analyses refer to the overall study population, including both normotensive and hypertensive individuals. This figure was specifically designed for this manuscript to juxtapose the relationships between arterial stiffness and clinic versus ambulatory hemodynamic variables, which were not presented as paired scatter plots in the original PAMELA publication (Cuspidi et al. *Clinical Research in Cardiology*, 2024 [23]).

The relationship between arterial stiffness and blood pressure components further refines this interpretation. As shown in Figure 2, arterial stiffness is associated, albeit weakly, with clinic systolic and diastolic blood pressure, whereas its relationship with ambulatory blood pressure appears more evident for the systolic component [23]. It should be noted that in the PAMELA cohort arterial stiffness was assessed using CAVI, which by design is less dependent on blood pressure at the time of measurement than conventional PWV [5,18]. This partly explains why the correlations with blood pressure components are weaker than those typically observed with carotid–femoral PWV in other settings [18]. Moreover, as arterial compliance declines, diastolic blood pressure progressively loses its relationship with vascular wall properties since it becomes increasingly dependent on heart rate and peripheral resistance rather than on elastic recoil [3]. This finding is physiologically coherent, as systolic pressure more accurately reflects the pulsatile component of hemodynamic stress [25]. When assessed over the 24 h cycle, systolic pressure captures repeated exposure to wave reflection and late systolic load, while diastolic pressure becomes less informative as arterial compliance declines and elastic recoil is reduced [11].

Blood pressure phenotype adds an additional layer of complexity. Masked and nocturnal hypertension are characterized by sustained hemodynamic stress outside the clinic and are consistently associated with a higher prevalence of target organ damage [27]. In these phenotypes, arterial stiffness may act as a multiplier, amplifying pulsatile stress during daily life and potentially accelerating myocardial and microvascular injury [28]. Isolated systolic hypertension provides a complementary model, often driven by vascular ageing, in which arterial stiffness plays a central role in shaping the hemodynamic profile and in promoting left ventricular hypertrophy [29]. Independent prospective data from other settings are largely in line with this interpretation. In the Kailuan cohort, a community-based study that included more than 40,000 participants and a mean follow-up of 5.5 years, higher brachial-ankle PWV was associated with incident heart failure in a graded, dose-dependent manner. This association remained significant even after adjustment for age, blood pressure, and conventional cardiovascular risk factors [30]. In a smaller but more extensively phenotyped study, Ali et al. reported a progressive increase in PWV from hypertensive controls to patients with established heart failure with preserved ejection fraction (HFpEF). In the same study, arterial stiffness was inversely related to peak exercise capacity and directly associated with echocardiographic markers of diastolic dysfunction [31]. Taken together, these observations, obtained in populations with different clinical characteristics and using different stiffness indices, support the view that the link between arterial stiffening and cardiac functional deterioration is not confined to a single measurement approach or clinical context [24,30,31].

In contrast, arterial stiffness shows no consistent relationship with heart rate in population-based analyses [23]. This apparent dissociation should not be interpreted as evidence against autonomic modulation, but it deserves further comment. Heart rate represents an imprecise surrogate of sympathetic neural activity [7] and reflects the net balance of sympathetic and parasympathetic inputs to the sinoatrial node, whereas arterial stiffness depends on vascular smooth muscle tone and structural wall properties, which are regulated by regional sympathetic vasoconstrictor outflow through a distinct effector pathway [7,32]. In population-based settings, where sympathetic activation tends to be mild and regionally heterogeneous, these two autonomic expressions may diverge substantially [7]. In a cohort of 88 healthy subjects spanning a wide age range, Holwerda et al. showed that muscle sympathetic nerve activity recorded by microneurography was an independent determinant of carotid–femoral PWV after adjustment for mean arterial pressure and heart rate, whereas heart rate itself added no predictive information to the model [33]. This confirms that heart rate and vascular sympathetic tone convey fundamentally different information, and that the absence of a clear relationship between heart rate and arterial stiffness in community-based analyses should not be taken as evidence against an autonomic contribution to vascular stiffening. This distinction becomes relevant when moving from community-based settings to more advanced cardiovascular conditions, in which autonomic activation is more pronounced and directly measurable [4].

Taken together, these findings indicate that, within a community-based setting, arterial stiffness identifies a subgroup of individuals in whom the hemodynamic burden of hypertension has already translated into early structural cardiac changes [25]. Importantly, this occurs even when left ventricular systolic function remains preserved and when conventional blood pressure measurements may not fully capture the extent of cardiovascular stress [34]. In this context, arterial stiffness reflects cumulative pulsatile load and ventricular exposure over time, rather than reflecting blood pressure levels at a single point [24].

## 5. Arterial Stiffness Across Hypertensive Phenotypes and Its Association with Cardiac Organ Damage

While the preceding section illustrated that arterial stiffening is already detectable in community-based populations before overt left ventricular systolic dysfunction becomes apparent [24,25], suggesting a temporal precedence of vascular over cardiac changes, this does not imply that vascular and cardiac alterations exist in isolation from each other. In cross-sectional analyses, individuals at different points along this trajectory coexist within the same sample, and those with more advanced vascular stiffening are also more likely to exhibit structural cardiac changes [35,36]. The data presented in this section should therefore be read as capturing different stages of a single progressive process at a given time point, not as contradicting the notion of temporal precedence.

Hypertension is increasingly recognized as a heterogeneous condition rather than a single hemodynamic entity [1,37]. Differences in blood pressure behavior across the 24 h cycle, in the relationship between office and out-of-office measurements, and in treatment response define distinct hypertensive phenotypes with different prognostic implications [38,39]. Within this framework, arterial stiffness provides a vascular dimension that helps reconcile apparently discordant clinical presentations, offering insight into why individuals with similar brachial blood pressure levels may experience markedly different patterns of target organ involvement [1,11].

**Figure 3:**
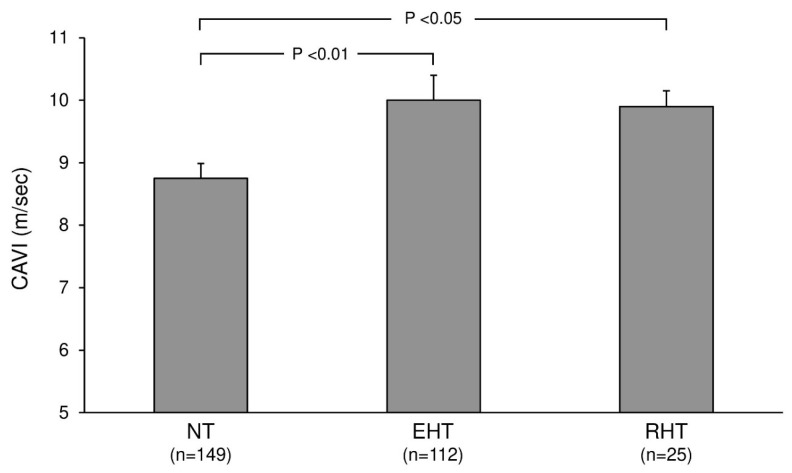
Arterial stiffness across normotension and hypertensive phenotypes. The figure illustrates cardio-ankle vascular index (CAVI) values across normotensive individuals and different hypertensive phenotypes, including essential hypertension and drug-resistant hypertension, within the Pressioni Arteriose Monitorate e Loro Associazioni (PAMELA) framework. This figure was developed for this manuscript to illustrate the distribution of arterial stiffness across multiple hypertensive phenotypes within a single comparative framework, an approach not adopted in the original PAMELA publications (Cuspidi et al. *American Journal of Hypertension*, 2024; 37:978–986 [24]).

When patients with hypertension are classified by blood pressure phenotype, arterial stiffness is consistently elevated across all phenotypes [37,39]. As shown in Figure 3, CAVI values increase progressively from normotensive individuals to those with essential hypertension and drug-resistant hypertension. Observations from other cohorts indicate that a similar pattern of increased arterial stiffness is also present in white coat, masked, isolated systolic, and nocturnal hypertension [37,39,40,41]. Thus, increased arterial stiffness may not be unique to a particular hypertension phenotype but rather a common underlying process that occurs independently of how blood pressure elevation manifests in routine clinical practice [36,39]. From a practical standpoint, this weakens the assumption that phenotypes traditionally considered less severe on the basis of office measurements are necessarily benign from a vascular perspective [37,38].

**Figure 4:**
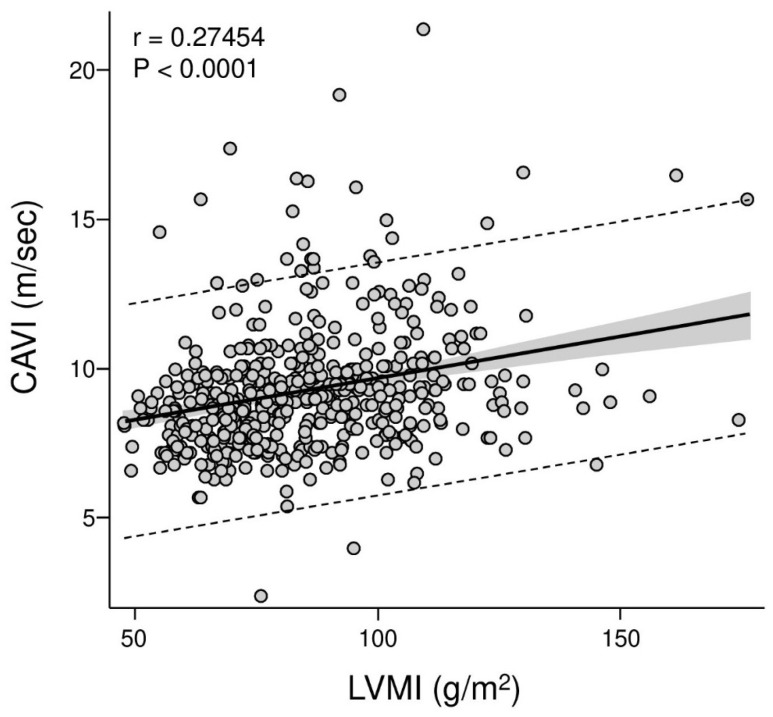
Association between arterial stiffness and left ventricular structural remodeling. The figure shows the relationship between cardio-ankle vascular index (CAVI) and left ventricular mass index (LVMI) in participants from the Pressioni Arteriose Monitorate e Loro Associazioni (PAMELA) cohort. This figure was created for this manuscript to isolate and visually emphasize the relationship between arterial stiffness and left ventricular structural remodeling, which in the source publication was reported within a broader multivariate context (Cuspidi et al. *Journal of Hypertension*, 2025; 43:781–789 [27]).

The relevance of this shared vascular substrate becomes more evident when arterial stiffness is examined in relation to cardiac organ damage [35]. As shown in Figure 4, arterial stiffness is directly associated with left ventricular mass index, indicating a close link between vascular properties and myocardial remodeling [35,42,43]. This association is consistent with a hemodynamic view of hypertension, in which increased stiffness of the large arteries amplifies the pressure load in late systole, thereby increasing the amount of pulse pressure transmitted to the left ventricle and resulting in hypertrophic changes in the myocardium, even in the absence of elevated blood pressure [10].

Interestingly, the relationship between arterial stiffness and left ventricular mass is seen in different blood pressure phenotypes, supporting a relationship that goes beyond a purely statistical association and is consistent with a mechanistic link, although cross-sectional data alone cannot establish causality [35,42]. Arterial stiffness modifies ventricular loading conditions in a manner that is biologically plausible and coherent with the development of hypertensive heart disease. In this context, stiffness may help explain the frequent dissociation between brachial blood pressure values and the extent of cardiac remodeling observed in clinical practice [44,45]. Patients with apparently adequate blood pressure control may nonetheless exhibit progressive structural changes, reflecting a persistent pulsatile burden that is not captured by conventional measurements [1,46].

Aside from left ventricular hypertrophy, arterial stiffness may be important in other forms of hypertension-mediated organ damage [15,47]. The facilitation of pulsatile pressure transmission to microvascular beds may contribute to organ damage in other organs, including the kidneys and brain, which often accompanies left ventricular remodeling [15,47]. Although these extracardiac manifestations are not the primary focus of the present review, their frequent association with increased arterial stiffness reinforces the concept of a systemic disease process rather than isolated organ involvement [46,47]. In this sense, arterial stiffness occupies a central position linking vascular, cardiac, and microvascular alterations across hypertensive phenotypes [1,46].

At this stage of the cardiovascular continuum, arterial stiffness serves as the bridge between the blood pressure phenotype and structural damage to the heart [48]. This is the pathophysiological setting in which the phenotypic classification of hypertension, based on blood pressure behavior, acquires mechanistic meaning and clarifies why organ damage may progress despite apparently satisfactory control of mean arterial pressure [45,46]. This perspective also helps frame the transition toward heart failure syndromes, because left ventricular hypertrophy and remodeling, particularly when accompanied by impaired relaxation and microvascular dysfunction, represent key substrates for subsequent clinical deterioration [48].

## 6. From Hypertensive Heart Disease to Heart Failure: Ventricular-Vascular Coupling as a Pathophysiological Bridge

The progression from hypertensive heart disease to heart failure represents an accumulation of changes in structure, function, and neurohumoral control [14,48,49]. In this context, ventricular–vascular coupling represents an important concept in understanding the interplay between vascular abnormalities and ventricular dysfunction [49,50,51]. When arterial compliance is preserved, the arterial tree acts as a buffer to pulsatile output, which is favorable to efficient ventricular performance [50]. However, as arterial stiffness increases, ventricular performance is progressively impaired, resulting in an increased rate of rise in systolic blood pressure and late systolic load on the left ventricle [49,51,52].

The implications of these changes on ventricular performance are far-reaching [50,51]. The increased late systolic load on the left ventricle leads to hypertrophy, which in turn increases myocardial oxygen demands, promoting subendocardial ischemia in the absence of significant coronary artery disease. The impaired ability to buffer pulsatile output also leads to decreased diastolic pressures, which can compromise coronary perfusion during exercise [50]. Ultimately, the net effect is a cascade of abnormalities in ventricular relaxation, leading to elevated left ventricular filling pressures and exercise intolerance [50].

These mechanisms are particularly relevant in HFpEF, where abnormal ventricular-arterial coupling, increased pulsatile load, and the coexistence of vascular and metabolic comorbidities are common [49,50]. In this context, the contribution of arterial stiffness to the increase in systolic load and the decrease in diastolic reserve would be expected to worsen the clinical manifestations [51]. Nevertheless, the impact of arterial stiffness is not limited to HFpEF [48]. In heart failure with reduced ejection fraction (HFrEF), coexisting vascular abnormalities are the rule rather than the exception, further impairing ventricular-arterial coupling efficiency and contributing to hemodynamic instability [50,52]. Apart from its impact on ventricular loading conditions, the pulsatile load transmitted by stiffened arteries may also affect the microcirculation and, consequently, the function of extracardiac organs [15,47]. In the setting of hypertensive heart disease and heart failure, renal and cerebral small vessel disease are common, and the coexistence of these conditions with the changes occurring in the ventricle suggests that the pathophysiological process is systemic rather than organ-specific [15,53]. In this regard, the common denominator between the changes occurring in the ventricle, the kidneys, and the brain would be the alterations in arterial stiffness [15,47]. Autonomic dysregulation is another layer of complexity in the pathophysiological picture, as the progression towards clinical heart failure is accompanied by activation of the autonomic nervous system, which, while initially compensatory, eventually becomes deleterious to both the ventricle and the vasculature [54]. Experimental and clinical data suggest that acute changes in autonomic tone, such as the increase in sympathetic tone, may have acute effects on arterial stiffness, acting on the tone of the vascular smooth muscle, while chronic changes may have long-term implications on the structure of the arterial wall [54].

Additional insights from patients with advanced heart failure provide further evidence of this interrelationship [54]. Under conditions where there is a high level of sympathetic activation, arterial stiffness is commonly elevated and closely related to indicators of ventricular dysfunction and neural sympathetic activity [54]. This supports the concept that, in the late stages of the disease, arterial stiffness is not simply a residue of previous damage but rather part of a complex, interrelated pathophysiological system that involves the heart, the vasculature, and the autonomic nervous system [48,54]. This interrelationship may not be apparent in the early stages of the disease, when autonomic nervous system activation is low and indirect markers of this activation, such as heart rate, do not accurately reflect the complex control of the autonomic nervous system [54].

Taken together, these factors place ventricular-vascular coupling at the center of the progression from hypertensive heart disease to heart failure [48,49,50]. Rather than a turning point, the development of heart failure might be viewed as a consequence of a progressive uncoupling that starts earlier in the course of hypertension [14,48,49]. From this perspective, arterial stiffness connects vascular ageing, myocardial remodeling, and neurohumoral activation from early hypertension to advanced heart failure [48,50,54].

## 7. Arterial Stiffness, Autonomic Dysregulation, and Advanced Heart Failure

As hypertensive heart disease progresses to the final stages of overt heart failure, the role of autonomic dysregulation becomes more pronounced and clinically significant [54]. Accordingly, the initially adaptive effects of sympathetic activation give way to a pathophysiological pattern that affects the course of the disease. In the final stages of heart failure, increased sympathetic activation is an established phenomenon and a major predictor of prognosis [55,56]. Another important but less emphasized aspect is the interrelation between the effects of autonomic activation and the properties of the arterial system, which may explain the pronounced arterial stiffening seen in the final stages of the disease.

There is evidence that sympathetic activation modulates arterial properties and that the effects of increased sympathetic drive on the arterial system may be both functional and structural [33]. Heightened sympathetic outflow may influence the smooth muscle contraction properties of the vessel wall and thereby modulate arterial compliance and PWV [57]. Moreover, sustained adrenergic stimulation may also induce structural changes in the arterial wall that may be associated with increased collagen deposition and decreased integrity of elastic fibers [33]. These observations are supported by a recent comprehensive review of cross-sectional and experimental human data, which concluded that the sympathetic nervous system modulates large artery stiffness through mechanisms that are at least partly independent of prevailing blood pressure levels, operating via direct effects on vascular smooth muscle tone and, over time, through structural remodeling of the arterial wall [32]. Therefore, in advanced disease, stiffness should be interpreted as the combined result of cumulative wall remodeling and contemporaneous neuro-adrenergic drive.

**Figure 5:**
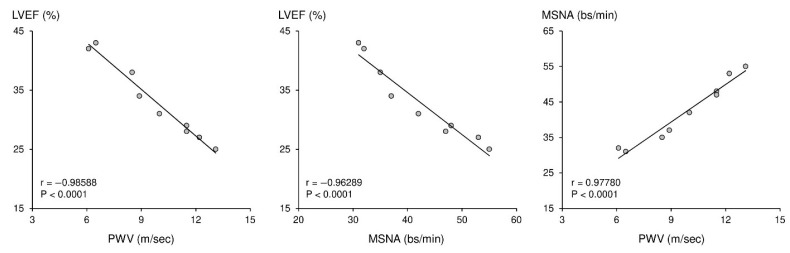
Relationships between arterial stiffness, sympathetic neural activity, and left ventricular systolic function in heart failure. The figure illustrates the associations between pulse wave velocity (PWV), left ventricular ejection fraction (LVEF), and muscle sympathetic nerve activity (MSNA) in patients with reduced ejection fraction enrolled in the Baroreflex Activation Therapy for Heart Failure (BATHF) program. This figure was originally designed for this manuscript to display in a single panel the triangular relationship among arterial stiffness, sympathetic neural activity, and left ventricular systolic function. This specific three-way visualization was not presented in the original BATHF publication (Gronda et al. *Clinical Research in Cardiology,* 2016; 105:838–846 [59]).

The BATHF program is a unique human model that may be used to explore this bidirectional relationship between autonomic dysregulation and arterial properties [8,58]. In the setting of advanced heart failure and reduced left ventricular ejection fraction, the unique opportunity to measure muscle sympathetic nerve activity provides a more accurate assessment of autonomic dysregulation than indirect markers such as heart rate. Baseline measurements in these patients with advanced heart failure and reduced left ventricular ejection fraction reveal a close interrelation among sympathetic neural activity, arterial properties, and left ventricular systolic dysfunction [8], and chronic baroreflex activation has been shown to favorably modify arterial stiffness in this setting [59]. Thus, as illustrated in Figure 5, increased PWV is strongly associated with reduced left ventricular ejection fraction and increased muscle sympathetic nerve activity.

These associations are pathophysiologically logical, as reduced cardiac output and diminished baroreceptor reflex function lead to increased sympathetic activity as a compensatory mechanism to maintain blood pressure. Increased sympathetic activity, in turn, is associated with increased arterial stiffness, which further increases ventricular afterload, thereby compromising ventricular-vascular coupling [33]. This establishes a self-perpetuating cycle in which sympathetic overactivity and arterial stiffening reinforce one another, accelerating hemodynamic deterioration.

A legitimate question is whether arterial stiffening in this context simply reflects the hemodynamic consequences of progressive ventricular failure and the resulting sympathetic overdrive, or whether sympathetic activation exerts a direct, independent effect on vascular mechanical properties. Evidence from acute physiological studies in humans favors the latter interpretation. Baroreceptor unloading through graded lower body negative pressure produces measurable increases in carotid-femoral PWV that cannot be accounted for by changes in mean arterial pressure alone, indicating that sympathetic outflow can stiffen central arteries through a mechanism distinct from the pressor effect itself [33]. Pharmacological ganglionic blockade in postmenopausal women reduces both PWV and augmentation index, even after correction for the concurrent blood pressure fall [32]. These experimental observations indicate that the sympathetic nervous system modulates arterial stiffness through a direct neurovascular pathway, operating alongside and partly independently of pressure-mediated wall stress. In advanced heart failure, where sympathetic overdrive is sustained and pronounced, this direct neural contribution probably compounds the structural stiffening produced by long-standing hemodynamic overload, establishing a self-reinforcing loop rather than a simple unidirectional causal sequence [54]. The BATHF data are consistent with this reading: the correlation between muscle sympathetic nerve activity and PWV was observed in patients with stable, optimally treated heart failure, a clinical setting in which acute hemodynamic decompensation was not the dominant driver of the associations observed [7,8].

It is noteworthy that the relationship between arterial stiffness and autonomic activity seems to be stage-dependent [54]. For population-based groups and early hypertensive phenotypes, arterial stiffness shows poor or inconsistent correlations with simple measures of autonomic function, such as heart rate. This lack of association may be explained by the limited sensitivity of indirect indices of autonomic function, as well as by the relatively mild degree of autonomic activation present in early disease states. By contrast, in more advanced heart failure states with marked sympathetic overactivity, a clear relationship with arterial stiffness is seen [8].

In addition to HFrEF, autonomic dysfunction and arterial stiffness are also relevant in HFpEF [60]. Although direct measures of sympathetic activity are less commonly available in this population, the coexistence of arterial stiffness, reduced ventricular–vascular coupling, and sympathetic overactivity supports a similar underlying pathophysiology, with arterial stiffness contributing to higher filling pressures and reduced exercise capacity [60], and being associated with worse prognosis in HFpEF [61].

Collectively, these findings highlight the role of autonomic dysregulation as a critical modulator of arterial stiffness in advanced cardiovascular disease [54]. Rather than being viewed as a discrete effect, the influence of sympathetic activation should be understood in the context of its interactions with vascular and cardiac dysfunction and across the spectrum of disease severity. Within the disease continuum from hypertension to heart failure, this reinforces the view of arterial stiffness as a phenotyping dimension that extends beyond cumulative vascular damage to encompass cardiac function and autonomic regulation [53].

## 8. Clinical Implications and Future Directions

The evidence reviewed in the preceding sections also has practical implications for clinical evaluation, some of which can already be placed within existing diagnostic pathways. One important point concerns the recognition of early cardiac structural abnormalities in individuals whose office blood pressure appears normal or adequately controlled. Observations from the PAMELA study and from other community-based cohorts [30,31] suggest that this apparent normality may be misleading in a meaningful proportion of cases, especially when out-of-office blood pressure is elevated but remains undetected. Under these circumstances, increased left ventricular mass or early diastolic abnormalities may already be present despite reassuring office measurements [25]. In this setting, combining ambulatory blood pressure monitoring, echocardiography, and arterial stiffness assessment offers a broader view of cardiovascular burden than office blood pressure alone. From a practical perspective, devices measuring CAVI or brachial–ankle PWV are commercially available, largely operator-independent, and suitable for outpatient use without substantial logistical complexity [19]. Their contribution may be particularly valuable in patients with isolated systolic hypertension, masked hypertension, or nocturnal hypertension, where the discrepancy between office blood pressure and actual hemodynamic load is often most evident [27], as well as in secondary hypertension, where subclinical vascular damage has been consistently reported [62].

Therapeutic implications are necessarily more cautious. No pharmacological treatment is currently approved with arterial stiffness reduction as a primary indication. Even so, several drug classes with well-established cardiovascular benefit have been shown to improve vascular properties in dedicated studies. Renin angiotensin system inhibitors and mineralocorticoid receptor antagonists, for example, can reduce PWV through mechanisms that are only partly explained by blood pressure lowering itself [1]. More recently, sodium–glucose cotransporter-2 (SGLT2) inhibitors have also shown favorable vascular effects, including reductions in PWV in patients with type 2 diabetes, although it remains uncertain whether these changes translate into a clinically relevant stiffness-specific benefit [63]. A similar argument applies to exercise-based rehabilitation. In both hypertension and heart failure, structured aerobic training has been associated with lower PWV together with improved functional capacity [64,65,66,67,68]. These interventions do not target arterial stiffness in isolation. Even so, the fact that vascular properties improve alongside broader cardiovascular adaptation supports the view that arterial stiffness should not be regarded simply as the passive expression of irreversible vascular damage, but rather as part of a disease trajectory that may still be modified [1].

At present, perhaps the most convincing clinical role of arterial stiffness lies in phenotypic refinement. Some patients show a degree of organ damage, exercise limitation, or incomplete response to treatment that seems disproportionate to the blood pressure values recorded in the clinic. In these cases, arterial stiffness assessment may help explain the mismatch and place apparently discordant findings within a more coherent pathophysiological framework [6]. This may be especially relevant in HFpEF, where altered ventricular vascular coupling, increased filling pressures, and reduced exercise tolerance coexist despite preserved systolic function [50]. In this context, arterial stiffness offers an integrative perspective that helps connect vascular, cardiac, and functional abnormalities within the same interpretive model [60]. A related consideration applies to HFrEF, in which ventricular dysfunction, arterial stiffening, and sympathetic activation frequently coexist, further supporting the view that the syndrome cannot be interpreted solely through impaired contractile performance [54].

Several methodological considerations should be kept in mind when interpreting the evidence discussed in this review. In the PAMELA cohort, arterial stiffness was assessed using CAVI, which reflects the properties of both elastic and muscular arterial segments and is therefore not directly interchangeable with carotid–femoral PWV, the current reference standard for aortic stiffness [16]. Comparative evidence further suggests that the prognostic performance of different stiffness indices may weaken as blood pressure is more extensively corrected, whereas conventional PWV appears to retain stronger discriminatory value in some settings [18,69]. In addition, the associations observed in PAMELA between arterial stiffness, blood pressure components, and left ventricular mass, although statistically significant, were modest in strength. This is not surprising in a community-based population with heterogeneous cardiovascular profiles, in which the contribution of any single hemodynamic variable is expected to explain only a limited proportion of overall variance [4]. By contrast, in the BATHF cohort, where disease severity is greater and autonomic activation is more pronounced, these relationships appear substantially tighter [7,8], in keeping with the stage-dependent vascular, cardiac, and neural interaction proposed in this review. It should also be acknowledged that the association between arterial stiffness and incident heart failure has not been observed consistently across all studies. In the Health, Aging, and Body Composition cohort, the relationship between carotid–femoral PWV and new-onset heart failure lost statistical significance after full adjustment for traditional cardiovascular risk factors [70]. This finding suggests that, particularly in older populations, the independent contribution of arterial stiffness may be difficult to disentangle from the broader burden of shared risk factors. For this reason, the framework proposed here should be interpreted as a pathophysiological model supported by converging observational and cross-sectional evidence, rather than as proof of a fully established causal sequence. Longitudinal studies with repeated vascular, cardiac, and autonomic assessments are still needed to clarify temporal ordering and to determine whether interventions that modify arterial stiffness also translate into measurable clinical benefit.

Future research should focus on longitudinal and interventional studies that incorporate vascular, cardiac, and autonomic endpoints. Of particular interest are approaches that assess whether modifying pulsatile hemodynamics, ventricular–vascular coupling, and autonomic activation can impact disease progression across the spectrum of hypertension and heart failure [49]. Ideal study designs would incorporate arterial stiffness measurements alongside imaging endpoints of remodeling and direct or indirect measures of sympathetic activation [54]. Such an integrated approach would address all the pathways discussed throughout this review.

## 9. Conclusions

Arterial stiffness occupies a strategic position in the continuum from uncomplicated hypertension to heart failure, linking the two conditions through its effects on pulsatile hemodynamics and ventricular–vascular coupling, regardless of hypertension phenotype and left ventricular function.

As cardiovascular disease progresses, the interaction between autonomic dysfunction and arterial stiffness becomes increasingly evident, particularly in advanced heart failure, where marked sympathetic activation is closely coupled with arterial stiffening. This observation underscores an integrated view of disease in which vascular, cardiac, and autonomic dysfunction are not separate events but part of one disease process.

In this context, arterial stiffness should no longer be considered as an end-result of cumulative damage. Rather, it should be recognized as a phenotyping dimension that encompasses the state of ventricular loading conditions, systemic vascular vulnerability, and autonomic balance. This view helps explain why cardiovascular risk and damage may persist despite well-controlled mean arterial pressure and why clinical phenotypes do not necessarily align with blood pressure values. Finally, this perspective raises the question of whether pharmacological strategies that favorably modify arterial properties, including but not limited to renin–angiotensin system inhibitors, mineralocorticoid receptor antagonists, and lipid-lowering agents [63,71], together with exercise-based rehabilitation approaches known to influence vascular function and exercise capacity [64,65,66,67,68], could translate into improved cardiovascular outcomes beyond blood pressure control. Answering this question will require dedicated interventional trials with arterial stiffness integrated as both a mechanistic endpoint and a potential therapeutic target.

## Data Availability

No new data were created or analyzed in this study. All data discussed are derived from previously published studies cited in the manuscript. All figures are original schematics conceived and designed by the authors specifically for this manuscript. They do not reproduce previously published figures and do not display novel analyses beyond what has been reported in the cited primary studies.

## Supplementary Materials

The following supporting information can be downloaded at: https://www.mdpi.com/article/10.3390/life16040682/s1, Supplementary Table S1: Relationship between manuscript figures and primary data sources. Each figure was originally conceived and designed by the authors for this manuscript. The table specifies the primary data source, the original analytical context in which the data were reported, and the specific element of visual synthesis added in the present work.
