# Supplementary material for: Beyond Blood Pressure: Arterial Stiffness as a Hemodynamic and Neuroadrenergic Axis Linking Hypertension, Cardiac Remodeling, and Heart Failure
Source: Life (Basel). 2026 Apr 16;16(4):682. doi: 10.3390/life16040682 (PMC13117860; doi:10.3390/life16040682)
Supplement: Supplementary file 1 [file life-16-00682-s001.zip › life-4229337-supplementary.pdf]

Supplementary Table S1. Relationship between manuscript figures and primary data sources. Each figure was originally conceived and designed by the authors for this manuscript. The table specifies the primary data source, the original analytical context in which the data were reported, and the specific element of visual synthesis added in the present work.

Figure	Primary data source	Original analytical context	What was presented in the source publication	What this figure adds
Figure 1	Cuspidi et al. *Clinical Research in Cardiology*. 2024 [23]	Population-based analysis of CAVI, blood pressure, and echocardiographic data in the PAMELA cohort	CAVI distributions, LVEF, LVEDD, and LVMI reported in separate tables and subgroup analyses	Joint visualization of four distributions in a single comparative panel across normotensive and hypertensive individuals
Figure 2	Cuspidi et al. *Clinical Research in Cardiology*. 2024 [23]	Correlational analysis of CAVI with blood pressure components and heart rate in the PAMELA cohort	Correlation coefficients reported numerically within a multivariate regression model	Integrated scatter plot panel comparing clinic versus ambulatory associations of CAVI with blood pressure components and heart rate side by side
Figure 3	Cuspidi et al. *American Journal of Hypertension*. 2024; 37:978-986 [24]	CAVI values stratified by blood pressure phenotype in the PAMELA cohort	Data reported in tabular form across phenotype subgroups	Visual comparison of CAVI across normotension, essential hypertension, and drug-resistant hypertension within a single schematic
Figure 4	Cuspidi et al. *Journal of Hypertension*. 2025; 43:781-789 [27]	Relationship between CAVI and left ventricular mass index in the PAMELA cohort	Association reported as part of a broader multivariate regression analysis	Isolated graphical representation emphasizing the CAVI–LVMI relationship as a standalone association
Figure 5	Gronda et al. *Clinical Research in Cardiology*. 2016; 105:838-846 [59]	Baseline hemodynamic and neural data in patients with advanced heart failure (BATHF program)	PWV, LVEF, and MSNA reported in separate tables and bivariate analyses	Triangular visualization linking PWV, LVEF, and MSNA in a single conceptual panel to illustrate the vascular–cardiac–neural axis

Abbreviations: CAVI, cardio-ankle vascular index; LVEF, left ventricular ejection fraction; LVEDD, left ventricular end-diastolic diameter; LVMI, left ventricular mass index; PWV, pulse wave velocity; MSNA, muscle sympathetic nerve activity; BATHF, Baroreflex Activation Therapy for Heart Failure.
